# Preparation of Cellulose-Based Flocculant and Its Application in the Enrichment of Vitamin K_2_ in Fermentation Supernatant

**DOI:** 10.3390/polym14122410

**Published:** 2022-06-14

**Authors:** Guoliang Ma, Zhiming Zheng, Han Wang, Li Wang, Genhai Zhao, Hengfang Tang, Xiumin Ding, Peng Wang

**Affiliations:** 1 Key Laboratory of High Magnetic Field and Ion Beam Physical Biology, Hefei Institutes of Physical Science, Chinese Academy of Sciences, 350 Shushanhu Road, Hefei 230031, China; ma1998@mail.ustc.edu.cn (G.M.); liwang@ipp.ac.cn (L.W.); zhgh327@ipp.ac.cn (G.Z.); hengfangt@outlook.com (H.T.); xmding@mail.ustc.edu.cn (X.D.); 2 Science Island Branch of Graduate School, University of Science and Technology of China, Hefei 230026, China; 3 CAS (Hefei) Institute of Technology Innovation Co., Ltd., Hefei 230088, China; 4 Anhui Key Laboratory of Environmental Toxicology and Pollution Control Technology, Hefei Institutes of Physical Science, Chinese Academy of Sciences, Hefei 230031, China

**Keywords:** cellulose, flocculant, *Bacillus subtilis natto* fermentation supernatant, vitamin K_2_

## Abstract

Nutritional food supplements and pharmaceutical products produced with vitamin K_2_ as raw materials a very promising market in the global scope. The main production method of vitamin K_2_ is microbial fermentation, but approximately 50% of vitamin K_2_ synthesized by the main production strain *Bacillus subtilis natto* exists in extracellular form, which is not easy to separate and extract. In order to solve this problem, in this study, we synthesized a novel cellulose flocculant, MCC-g-LMA, by grafting reaction using microcrystalline cellulose (MCC) and lauryl methacrylate (LMA) as monomers, and ammonium persulfate as an initiator to flocculate VK_2_ from the fermentation supernatant. The flocculant was characterized by Fourier transform infrared spectroscopy (FTIR), elemental analysis, and scanning electron microscopy (SEM), and the grafting reaction was successful. When the flocculant dosage was 48.0 mg/L and pH was 5.0, the flocculation rate of the MCC-g-LMA on the fermentation supernatant reached 85.3%, and the enrichment rate of VK_2_ reached 90.0%. Furthermore, we explored the flocculation mechanism of VK_2_ by the MCC-g-LMA and speculated that the flocculation mechanism mainly included adsorption bridging, hydrophobic association and net trapping and sweep effect. In this study, the extraction method for trace high-value biological products in the fermentation supernatant was improved, which provided a method and theoretical basis for the efficient separation and purification of VK_2_ and other terpenoids.

## 1. Introduction

The efficient and economical extraction of trace high-value biological products from fermentation supernatant has always been a hot topic [1,2]. As a fat-soluble vitamin, vitamin K_2_ (VK_2_) has a good effect on mitochondrial damage repair, and the prevention and treatment of osteoporosis, and cardiovascular and neurodegenerative diseases [3,4]. Currently, it is recommended by the European Food Safety Authority for dietary supplements and the clinical treatment of several diseases, with a large market demand. Due to the limitations of the chemical synthesis of VK_2_ and environmental pollution problems [5], an increasing number of scholars prefer to use microbial fermentation to obtain VK_2_ with high activity [6].

The main production strain of VK_2_ is *Bacillus subtilis natto*. After fermentation, VK_2_ mainly exists in bacteria and the fermentation supernatant, and its content in the fermentation supernatant accounted for about 50% of the total. However, the content of VK_2_ in the supernatant of the fermentation supernatant is very low, which hinders extraction and enrichment. At present, a large amount of n-hexane, isopropanol and other solutions are required to extract VK_2_. This method has disadvantages, such as the large consumption of organic reagents, high cost, and environmental pollution, which render it almost impossible to apply in large-scale industrial production [7]. VK_2_ secreted by *Bacillus subtilis natto* in a fermentation supernatant was reported to form a water-soluble macromolecular compound by combining with an amphiphilic polypeptide and dispersing it in the fermentation supernatant [8]. This study provides the possibility of extracting VK_2_ secreted by *Bacillus subtilis natto* with flocculants.

Cellulose has huge reserves and wide sources in nature. As a natural flocculating material, it has been used in sewage treatment, pulp production, metal extraction, and other industries. In addition, cellulose has good biocompatibility and easy degradability, and many studies reported that cellulose has been used to produce innovative biodegradable or functional materials [9,10,11]. So, it has good application prospects in the enrichment and extraction of biological products [12,13], However, studies have shown that, when natural cellulose is directly used as the adsorption material, its adsorption capacity and selectivity are very low because a large number of hydroxyl groups exist in the structure of cellulose polymer, and hydrogen bonds are extensively formed between and within the molecular chains, affecting its reaction activity [14]. The direct modification method can be used to directly introduce functional groups into the cellulose framework as the binding sites between suspended substances and colloidal particles to prepare cellulose compounds, and improve the adsorption capacity and selectivity of materials [15,16,17,18]. Wei et al. used acrylic acid and lauryl methacrylate (LMA) to prepare an amphiphilic flocculant that was used to settle soluble organic matter in water [19]. Wu et al. used LMA to prepare a polymer material that could absorb various fat-soluble compounds in seawater [20]. Acrylate compounds have good absorption capacity for lipid-soluble compounds [21]. If such functional groups are introduced into cellulose-based flocculants, it is possible to improve the extraction of VK_2_ in the fermentation supernatant.

On the basis of the above research, we attempted to graft LMA onto the surface of microcrystalline cellulose to prepare a novel cellulosic flocculant, MCC-g-LMA, to flocculate VK_2_ in the fermentation supernatant of *Bacillus subtilis natto*. In addition, we comprehensively characterized the structure of the flocculant, performed a single-factor analysis test, determined the key factors, optimized the flocculation process parameters, and efficiently flocculated VK_2_ in a fermentation supernatant. Moreover, the main mechanism of VK_2_ enrichment by the MCC-g-LMA was investigated, which provided a basis for the efficient separation, purification, and industrial production of VK_2_ and other terpenoids.

## 2. Materials and Methods

### 2.1. Materials

Microcrystalline cellulose (MCC), ammonium persulfate ((NH_4_)_2_S_2_O_8_), anhydrous calcium chloride (CaCl_2_), diatomite, and other experimental materials were purchased from Shanghai Sangong Biological Engineering Co., Ltd. (Shanghai, China). Lauryl methacrylate (LAM), urea (CH_4_N_2_O), sodium hydroxide (NaOH), naphthoquinone (menadione), and squalene (C_30_H_50_) were purchased from Shanghai Aladdin Biochemical Technology Co., Ltd. (Shanghai, China). Vitamin K_2_ (MK-7) standard (purity and GT; 99%), methanol, dichloromethane, acetonitrile and isopropanol chromatographic pure solvents were purchased from Sigma Company in the United States (St. Louis, MO, USA); ethanol, n-hexane, n-butanol, dichloromethane, isopropanol and other analytical solvents were purchased from Sinophem Chemical Reagent Co., Ltd. (Shanghai, China).

We used electronic analytical balance BSA124S (Sartorius, Beijing, China), temperature-controlled magnetic stirrer JB-3 (Jarrel electric, Jintan, China), Fourier transform infrared spectrometer ALPHA-T (Bruker, Karlsruhe, Germany), Elementar Vario EL Cube (Elementar, Hanau, Germany), high-performance liquid chromatographer 1260 Infinity (Agilent, Palo Alto, CA, USA), scanning electron microscope GeminiSEM 500 (Zeiss, Oberkochen, Germany), and frozen centrifuge Avanti J-E (Bechman, Brea, FL, USA).

### 2.2. Preparation of Cellulose Grafted Lauryl Methacrylate Flocculant (MCC-g-LMA)

First, microcrystalline cellulose (2 g) was dissolved in prefrozen 5 wt% NaOH/11 wt% urea solution at −20 °C to obtain a transparent cellulose solution that was added to a four-mouth flask with nitrogen access equipment, a thermometer, an agitator, and a condenser, and stirred with nitrogen at a certain temperature for 40 min. Then, ammonium persulfate solution (2 mg/mL) was added for 20 min, and the water phase of the whole reaction system was no more than 40 mL. Lauryl methacrylate (4 g) was added and heated to 95 °C for approximately 1.5 h to form a gelatinous substance.

### 2.3. Characterization

Microcrystalline cellulose (MCC) and cellulosic flocculant (MCC-g-LMA) were characterized by using elemental analysis (EA), Fourier transform infrared (FTIR) spectroscopy, and field-emission scanning electron microscopy (FESEM). Fourier transform infrared (FTIR) spectra were recorded on a Bruck Alpha-t FTIR spectrometer. Lyophilized samples were mixed with potassium bromide for analysis, and after 64 consecutive scans, spectra were collected at 4 cm^−1^ resolution. The samples prepared in advance were placed into scanning electron microscope GeminiSEM 500 to directly observe and study the surface morphology of the samples. Elemental analysis (Vario Micro Cube, Elementar, Germany) was used to determine the content % of carbon (%C), nitrogen (%N), hydrogen (%H), and oxygen (%O) in the samples.

### 2.4. Flocculation Experiment

After the preparation of the flocculant, its performance should be tested first. The flocculation capacity of microcrystalline cellulose (MCC) and cellulosic flocculant (MCC-g-LMA) was tested by the centrifugation of the *Bacillus subtilis natto* fermentation supernatant. First, six 50 mL fermentation supernatants of *Bacillus subtilis natto* whose OD value and VK_2_ concentration were tested were placed in a brown beaker. Three fermentation supernatants were used as a set of parallel experiments, with the sequence of Nos. 1, 2 and 3. The fermentation supernatants was stirred for 5 min in advance. Then, 50 mg of anhydrous CaCl_2_ was added and stirred for 10 min. The same amounts of microcrystalline cellulose and MCC-g-LMA were added to different beakers. The suspension was stirred at 250 rpm for 40 min, and then mixed slowly at 50 rpm for 20 min. Lastly, 1 g diatomite was added as a filter aid, and the suspension was centrifuged at 1500 rpm for 5 min to leave the precipitate. At the end of the centrifugal sedimentation time, the OD value of the supernatant was recorded, and the flocculation rate was calculated. The specific calculation method is shown in Formula (1).

Flocculation rate = (OD_1_ − OD_2_)/OD_1_ × 100% (1)

where OD_1_ denotes the OD value of the *Bacillus subtilis natto* fermentation supernatant before flocculation supernatant, and OD_2_ denotes the OD value of the *Bacillus subtilis natto* fermentation supernatant broth after flocculation.

### 2.5. Drawing of Standard Curve and Determination of Vitamin K_2_

The VK_2_ (MK-7) standard was accurately weighed, and methanol was used as solvent to prepare VK_2_ standard solution with concentrations of 120, 110, 90, 70, 50, 30, 20, and 10 mg/L. VK_2_ was filtered through a 20 μm organic membrane and detected by Agilent high-performance liquid chromatography (HPLC). The mobile phase consisted of methanol/dichloromethane mixture (1:4, *v*/*v*) at a flow rate of 1.0 mL/min. The UV wavelength was 248 nm, and the column temperature was 35 °C.

### 2.6. Determination of Enrichment Rate of Flocculant for Vitamin K_2_

After flocculation performance experimental testing, each sample of flocculation precipitation was collected, and after dealing with the flocculation of clear liquid fermentation, a certain amount of anhydrous ethanol extraction was used in the flocculation experiment to obtain the settlement of VK_2_. An n-hexane/isopropanol/n-butyl alcohol mixture was used in the clear liquid extractive fermentation to remove VK_2_. After extraction, the content of VK_2_ in the flocculation sedimentation product and the remaining amount of VK_2_ in the fermentation supernatant after flocculation were calculated, and the enrichment rate was lastly calculated. The specific calculation method is shown in Formula (2).

Enrichment rate = (M_0_ − M_1_)/M_0_ × 100% (2)

where M_0_ denotes the total mass of VK_2_ in the fermentation supernatant before flocculation in mg, while M_1_ denotes the remaining mass of VK_2_ in the fermentation supernatant after flocculation in mg.

### 2.7. Determining the Influence of Dosage of Flocculant on Flocculation Effect

Six groups of parallel experiments were prepared. Eight 20 mL fermentative supernatants with measured OD values and VK_2_ concentrations were weighed in each group. Microcrystalline cellulose (MCC) was added in Groups 1, 2, and 3, and cellulosic flocculant (MCC-g-LMA) was added in Groups 4, 5, and 6. The flocculant dosage in each group was 4, 8, 16, 24, 32, 40, 48, and 56 mg. The effects of different dosages of MCC and MCC-g-LMA on the flocculation rate and VK_2_ enrichment rate were compared. After adding different kinds and amounts of flocculants, the suspension was stirred at 250 rpm for 40 min, and then mixed slowly at 50 rpm for 20 min. Lastly, 1 g diatomite was added as a filter aid, and the suspension was centrifuged at 1500 rpm for 3–5 min to leave the precipitation. After the OD value of each supernatant had been recorded, VK_2_ in the precipitation was extracted, and the flocculation rate and VK_2_ enrichment rate were calculated.

### 2.8. Determining the Influence of pH on Flocculation Effect

Six groups of parallel experiments were also prepared. In each group, 6 samples of 20 mL fermentation supernatant with measured OD values and VK_2_ concentrations were weighed, and the 6 samples of fermentation supernatant in each group were configured with pH values of 4, 5, 6, 7, 8 and 9. Groups 4, 5, and 6 were added with cellular flocculant (MCC-g-LMA), and the addition amount of microcrystalline cellulose (MCC) and cellular flocculant (MCC-g-LMA) was 48 mg per sample to explore the influence of pH on the flocculation effect. The specific operations of the following steps are basically consistent with those in Section 2.7.

### 2.9. Study on the Mechanism of VK_2_ Enrichment by MCC-g-LMA

In addition to the above experiments, we also partially explored the mechanism of vitamin K_2_ enrichment by flocculant. First, 50 mg vitamin K_3_ and 10 mg squalene were added to 100 mL ultrapure water and stirred at 250 rpm for 10 min to form a uniform vitamin K_3_ suspension solution and squalene suspension system. An appropriate amount of flocculant was added to the suspension and stirred at 250 rpm for 40 min; then, diatomite was added as a filter aid for 1 g, centrifuged at 1500 rpm for 3–5 min, and the precipitation was left. The enriched vitamin K_3_ and squalene in the precipitation of the two systems were each extracted with the mixed solution of n-hexane/isopropanol/n-butanol. Vitamin K_3_ and squalene were determined by high-performance liquid chromatography. An acetonitrile/water (70/30, *v*/*v*) solution was used as the mobile phase in the HPLC test with a flow rate of 0.85 mL/min. The detection wavelength was 265 nm, and the column temperature was 40 °C [22]. The mobile-phase system of methanol/isopropanol (70/30, *v*/*v*) was adopted for the detection of squalene by HPLC. The detection wavelength was 205 nm, the flow rate was 1 mL/min, and the column temperature was 30 °C [23]. After the test, the enrichment rate of vitamin K_3_ and squalene by the flocculant was calculated by using the standard curve.

## 3. Results and Discussion

### 3.1. Preparation of Flocculant

**Figure 1:**
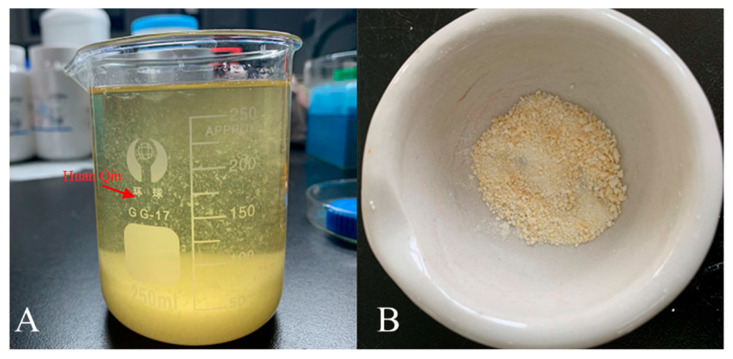
Washing and drying of flocculated products. (**A**) Flocculant washing process; (**B**) flocculant after drying.

The colloidal products were washed three times with an ethanol solution (70%) and then washed with ultrapure water. The washing process is shown in Figure 1A. After natural drying, a MCC-g-LMA solid was obtained that was ground into powder, sealed for preservation, and used as the experimental flocculant. The flocculant powder is shown in Figure 1B.

### 3.2. Characterization of MCC-g-LMA

#### 3.2.1. FTIR Spectra

**Figure 2:**
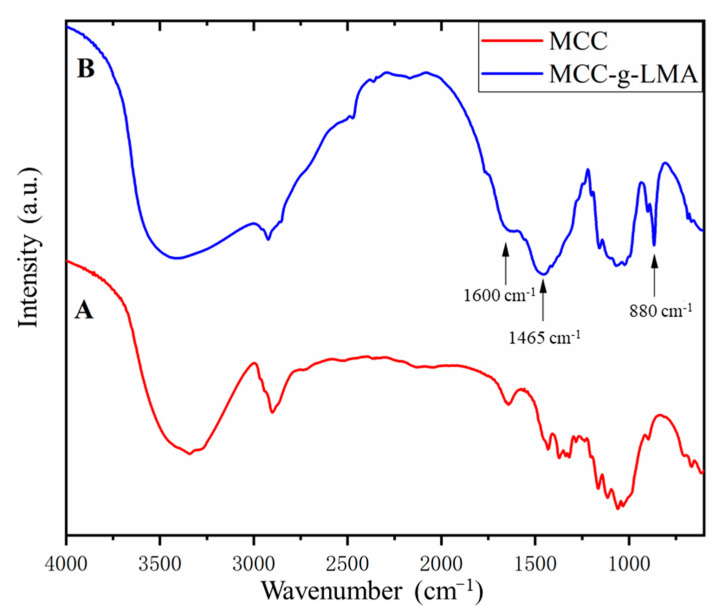
FTIR spectra of MCC and MCC-g-LMA. (**A**) MCC; (**B**) MCC-g-LMA.

MCC-g-LMA and microcrystalline cellulose (MCC) samples were dried and ground into fine powder with potassium bromide for Fourier transform infrared spectroscopy (FTIR). Results show that the proportion of the LMA in the reaction product was effectively increased. The NIR characteristic absorption peaks of some functional groups of flocculant are shown in Table 1. Figure 2A shows the FTIR spectrum of the MCC. Wavenumber 3400 cm^−1^ is the infrared characteristic absorption peak of hydroxyl (O–H), and wavenumber 1020 cm^−1^ is the infrared characteristic absorption peak of the –C–O– bond, indicating that the contents of hydroxyls and –C–O– bonds were high in MCC [12]. Figure 2B shows the FTIR spectra of the MCC-g-LMA. Wavenumber 880 cm^−1^ in the figure shows the stretching vibration absorption peak of the R_2_C=CHR functional group bond, which is unique to LMA. The wave at 1020 cm^−1^ was the stretching vibration absorption peak of the –C–O– functional group bond, showing a significant increase in the number of –C–O– functional groups. Wavenumber 1465 cm^−1^ is the stretching vibration absorption peak of the –CH_2_– functional group bond. The absorption peak of MCC-g-LMA was higher than that of MCC because the proportion of –CH_2_– bonds was higher in the flocculant of LMA with a long carbon chain. The new peak with a wavenumber of 1660 cm^−1^ was the absorption peak of ester carbonyl (C=O), which is unique to LMA. These results prove that LMA was added to the main chain of cellulose [19,24,25].

#### 3.2.2. SEM Analysis

**Figure 3:**
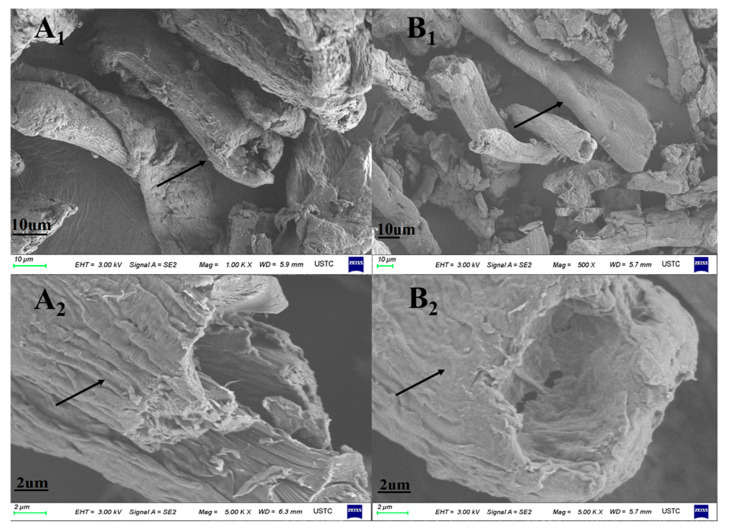
SEM micrographs of (**A_1_**,**A_2_**) MCC and (**B_1_**,**B_2_**) MCC-g-LMA. The position indicated by the arrow represents the characterization difference of different flocculants under SEM.

FTIR results were verified with scanning electron microscopy (SEM) observations. The surface morphology of MCC and MCC-g-LMA was studied with SEM. The SEM photographs of the MCC and MCC-g-LMA are shown in Figure 3, and specific experimental details of SEM analysis are shown in Table 2. The SEM analysis of MCC and MCC-g-LMA shows that the MCC had a rodlike multigap and multilayer structure, and a rough surface. After grafting, the structure of the MCC-g-LMA still had rodlike and multigap characteristics, but the surface became relatively smooth, and the layers were no longer distinct. SEM analysis shows that the original structure of the MCC was changed by polymerization, surface smoothness was improved, and the gap structure still existed with a considerable specific surface area [26].

#### 3.2.3. Elemental Analysis

On the basis of the elemental analysis of MCC and MCC-g-LMA, the contents of nitrogen, oxygen, and carbon in the MCC and MCC-g-LMA are shown in Table 3. According to the molecular formulas of MCC and LMA, the content of the C element in MCC was lower than that in LMA, and the content of the O element in MCC was higher than that in LMA. Whether the LMA is directly attached to the cellulose chain can be determined by observing the contents of the C and O elements in the grafted product. Results show that the contents of C, O and H elements in the grafted product significantly increased, verifying the successful grafting of the LMA onto the main chain of cellulose.

### 3.3. Flocculation Performance Experiment

Two groups of parallel experiments were set. MCC and MCC-g-LMA were separately added to the fermentation supernatants of the two groups, and flocculation rates were calculated. Results are shown in Table 4. Compared with MCC, MCC-g-LMA significantly increased the flocculation rate of the fermentation supernatant. MCC could be used for flocculation because a small number of colloidal particles contains a small amount of cellulose surface hydroxyls and other active groups, and some metal ions and organic compounds can be adsorbed on the active sites. However, due to the formation of a relatively large number of molecule-to-molecule hydrogen bonds within the hydroxyls on its molecular chain, the reactivity of other active groups is not high [15], leading to a poor flocculation effect.

### 3.4. Vitamin K_2_ Standard Curve Drawing and Content Determination

**Figure 4:**
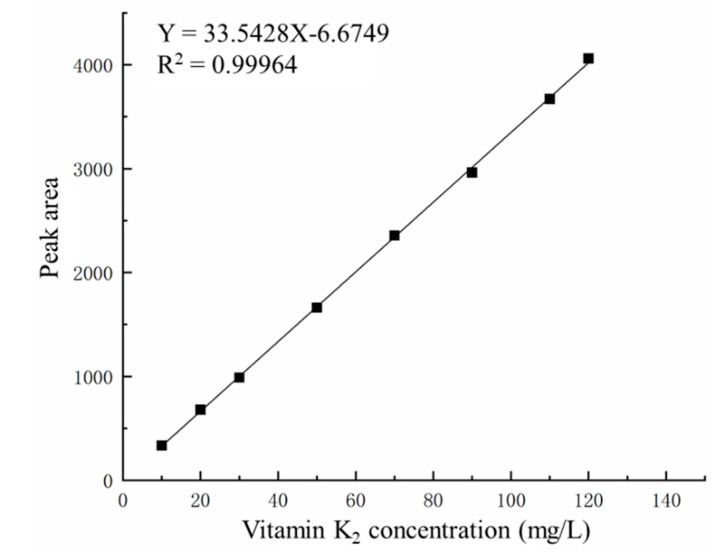
Standard curve of vitamin K_2_.

The sample of each standard solution was tested by high-performance liquid chromatography (HPLC). Then, the peak areas of the standard solutions with different concentrations were recorded to draw the standard curves. Results are shown in Figure 4. The curve fitting equation was Y = 33.5428X − 6.6749, and R^2^ = 0.99964.

### 3.5. Extraction and Enrichment of Vitamin K_2_ from Flocculation Sedimentation Products

Ethanol was used to extract VK_2_ from the fermentation supernatant after flocculation treatment and flocculation precipitation. HPLC was used to detect the content of VK_2_ in the extraction solution of each sample. Then, enrichment rates were calculated, and results are shown in Table 5. According to the experimental results, after treatment with MCC-g-LMA, nearly 70% VK_2_ in the fermentation supernatant could be enriched, proving the good enrichment effect of the newly prepared flocculant on VK_2_ in the fermentation supernatant.

### 3.6. Influence of Dosage of Flocculant on Flocculation Effect

**Figure 5:**
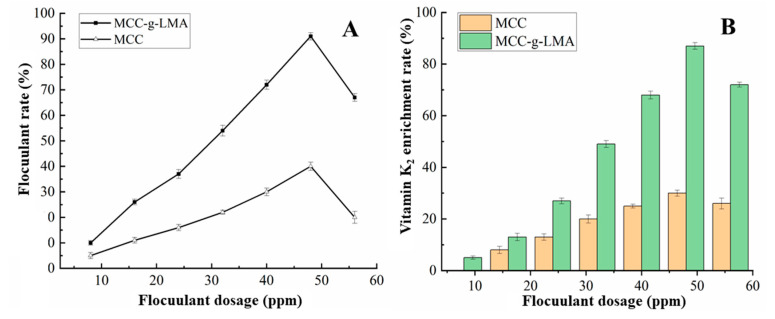
Effect of flocculant dosage on (**A**) flocculation rate and (**B**) the enrichment rate of vitamin K_2_.

The fermentation supernatant of *Bacillus subtilis natto* was flocculated with CaCl_2_ as a coagulant, and MCC and MCC-g-LMA as flocculants. Relevant studies show that the amount of CaCl_2_ used is 300.0 mg/L, which is pretreated to promote coagulation [27]. A parallel experiment was prepared. In each experiment, 20 mL of fermentation supernatant was weighed, and the dosages of the flocculant were 4, 8, 16, 24, 32, 40, 48, and 56 mg. Figure 5A shows the flocculation rates of different dosages of MCC or MCC-g-LMA on the fermentation supernatant of *Bacillus subtilis natto*. The figure shows that the flocculation rate first increased and then decreased with the increase in flocculant dosage after reaching the peak value. This phenomenon is consistent with the bridging flocculation mechanism, and the increase in flocculant dosage is conducive to full bridging with the particles. When the flocculant covered nearly 50% of the particle surface, the flocculation effect was the best [28]. However, an excessive dosage causes adsorption saturation and the formation of a covering layer on each colloidal particle so that the colloidal particles are stabilized again. Therefore, too-high or too-low flocculant dosages in the system lead to poor flocculation effects. We added 1 g of diatomite as a filter aid, centrifuged the flocculating products, measured the contents of VK_2_ in the flocculating products and the supernatants of the fermentation supernatant, and compared the effects of the flocculant dosages on the enrichment rates of VK_2_. Results are shown in Figure 5B. With the increase in flocculant dosage, the concentrations of VK_2_ in the fermentation supernatant first increased and then decreased, which may have been related to the flocculant dosage related to the influence of the flocculating rate in the fermentation supernatant. When the dosage of the flocculant was 48 ppm, the enrichment rate of VK_2_ in the supernatant of fermentation was the highest, which could reach more than 90%. The main form of VK_2_ in the fermentation supernatant is a colloidal complex with other protein substances, and the increase in flocculation rate leads to the increase in colloidal particles such as protein and VK_2_, so the enrichment rate of VK_2_ changes with the change in flocculation rate.

### 3.7. Influence of pH on Flocculation Effect

**Figure 6:**
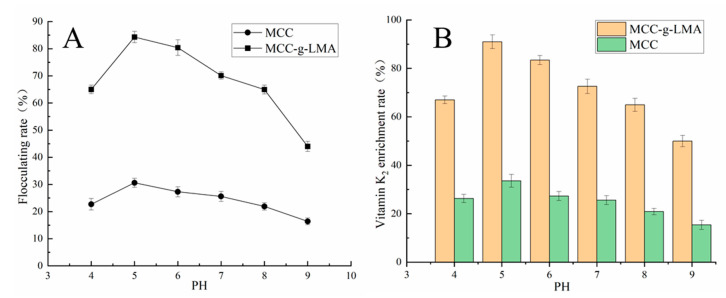
Effect of pH on (**A**) flocculation rate and (**B**) the enrichment rate of vitamin K_2_.

In the preliminary stage, we performed the flocculation experiments under the conditions of pH > 4.0 and pH > 9.0 of the fermentation supernatants. Results show that the flocculation effect was poor, which may have been because the molecular extension and dissolution of the flocculant were affected when the pH was relatively low or high. Figure 6 shows that, within the range of pH 4.0–9.0, the flocculation effect was the best, with a flocculation rate of approximately 85.3% and an enrichment rate of VK_2_ of 90% when the pH of the flocculation system was 5.0.

### 3.8. Discussion on the Mechanism of Vitamin K_2_ Enrichment with Flocculant

**Figure 7:**
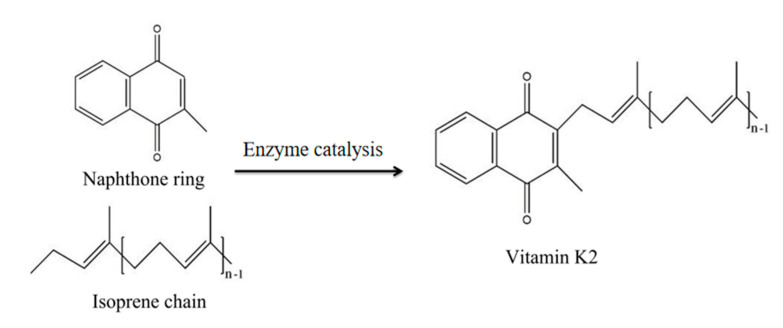
Structure and composition of vitamin K_2_. n-1: the number of isoprene units.

**Figure 8:**
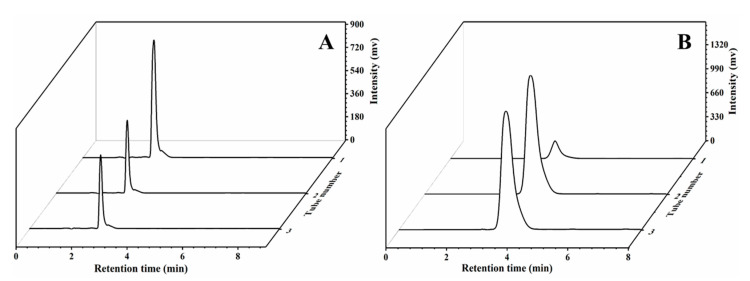
HPLC chromatograms of the collected samples from menadione and squalene simulation system by RP-C18 chromatography: (**A**) MCC-g-LMA effect of the flocculant on menadione; (**B**) MCC-g-LMA effect of the flocculant squalene.

VK_2_ in bacteria is mainly enriched through the flocculation of bacteria, and this mechanism has been fully studied. However, there are few studies on the mechanism of extracellular VK_2_ adsorption. Through the survey, we found that the LMA used in the experiment had been used in the oil absorption material, and the prepared acrylic resin could adsorb and hydrophobically associate to fix some fat-soluble compounds. Therefore, we explored whether MCC-g-LMA had adsorption and hydrophobic association effects on VK_2_, which is a fat-soluble compound. VK_2_ is a type of terpene composed of naphthoquinone rings and isoprene side chains. As shown in Figure 7, in this section, we selected vitamin K_3_ (VK_3_), which is only composed of naphthoquinone rings, and squalene, which is only composed of isoprene side chains, as the research objects to explore the enrichment effect of MCC-g-LMA on naphthoquinone rings and isoprene side chains, respectively [29,30]. Figure 8A shows the enrichment effect of the flocculant on VK_3_ containing the naphthoquinone rings only. Results show that the enrichment rate of the flocculant on the naphthoquinone rings was approximately 45% after flocculation. Figure 8B shows the enrichment effect of the flocculant on squalene containing isoprene side chains only, illustrating that the enrichment rate of the flocculant on isoprene side chains is approximately 65% after flocculation. These results indicate that MCC-g-LMA had a hydrophobic association effect on the naphthoquinone rings and isoprene side chains, thus enriching VK_2_ in the fermentation supernatant. Moreover, the flocculant could flocculate polypeptide or protein molecules containing VK_2_ by adsorption bridging, and the relatively large flocs could be further strengthened by the net trapping and sweep.

## 4. Conclusions

In this study, a novel cellulosic flocculant, MCC-g-LMA, was synthesized by the grafting reaction of MCC and LMA as monomers. MCC-g-LMA was characterized by elemental analysis, FTIR, and SEM. The results of structural characterization show that the preparation was successful. Compared with traditional cellulose, the flocculation effect of MCC-g-LMA on the fermentation supernatant was remarkable. When the flocculant dosage was 48.0 mg/L and the pH was 5.0, the flocculation rate of the *Bacillus subtilis natto* fermentation supernatant could reach 85.3%, and the enrichment rate of VK_2_ in the fermentation supernatant could reach more than 90%.

**Figure 9:**
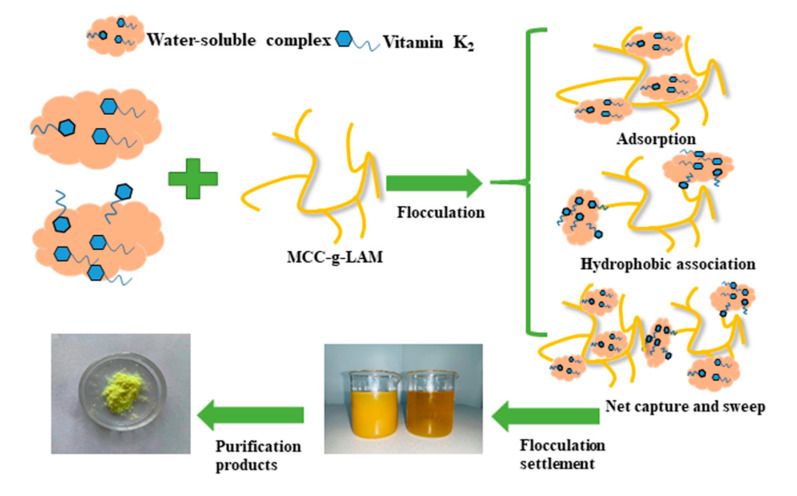
Schematic diagram of flocculation mechanism of MCC-g-LMA.

In this study, the enrichment effect and main mechanism of the novel flocculant on the VK_2_ in fermentation supernatant were investigated. Using VK_3_ containing naphthoquinone rings in VK_2_ only and squalene containing isoprene side chains in VK_2_ only as the flocculation objects, the enrichment effect of MCC-g-LMA was explored. Results show that the enrichment rates of MCC-g-LMA on naphthoquinone rings and isoprene side chains were approximately 45% and 65%, respectively. We speculated that the main enrichment methods for VK_2_ in the fermentation supernatant by the MCC-g-LMA were as follows: (1) MCC-g-LMA played an adsorption bridging role on the compounds formed by VK_2_ and polypeptide molecules, and then enriched the VK_2_; (2) MCC-g-LMA had a hydrophobic association effect on naphthoquinone rings and isoprene side chains, thus enriching the VK_2_ in fermentation supernatant; (3) MCC-g-LMA formed large flocs in the flocculant, and enriched the VK_2_ in the fermentation supernatant through the net trapping and sweep effect. Figure 9 shows the flocculation mechanism of MCC-g-LMA.

In this study, a green and efficient extraction method for extracellular VK_2_ from *Bacillus subtilis natto* was designed using a flocculant and a safe solvent, ethanol, which eliminated the previous limitation of liquid-liquid extraction of VK_2_ from fermentation supernatants using toxic solvents. The prepared flocculant was environmentally friendly and had good flocculation performance. This study provided theoretical and methodological basis for the efficient separation and purification of terpenoids such as VK_2_. At present, the new flocculant can only extract about 90% of VK_2_ from the fermentation supernatant. In the next work, we plan to design several different bases of flocculant, continue to optimize the flocculation conditions, and try to achieve the one-step extraction of all intracellular and extracellular VK_2_ from a fermentation liquid.

## Figures and Tables

**Table 1:** Infrared characteristic absorption peaks of different functional groups.

Functional Groups	Characteristic Peak of IR Spectrum	Functional Groups	Characteristic Peak of IR Spectrum
O–H	3400 cm^−1^	R_2_C=CHR	880 cm^−1^
–CH_2_–	1465 cm^−1^	C=O	1660 cm^−1^

**Table 2:** SEM specific parameters.

		Working Distance	Beam Energy	Magnification
SEM Order				
A_1_		5.9 mm	3.0 kV	1.0 K ×
A_2_		6.3 mm	3.0 kV	5.0 K ×
B_1_		5.7 mm	3.0 kV	500 ×
B_2_		5.7 mm	3.0 kV	5.0 K ×

**Table 3:** Elemental analysis results of MCC and MCC-g-LMA.

	Element Content	%N	%C	%H	%O
Sample					
MCC		0.04	40.23	5.919	50.887
MCC-g-LMA		0.03	47.68	7.137	44.794

**Table 4:** Experimental results of flocculation performance of MCC and MCC-g-LMA.

	Sample Number	1	2	3
Flocculation Rate (%)				
MCC		26.7%	25.3%	30.1%
MCC-g-LMA		72.4%	71.3%	69.2%

**Table 5:** Extraction and enrichment of vitamin K_2_ from MCC and MCC-g-LMA flocculation products.

	Sample Number	1	2	3
Flocculation Rate (%)				
MCC		26.7%	25.3%	30.1%
MCC-g-LMA		72.4%	71.3%	69.2%

## Data Availability

The data presented in this study are available on request from the corresponding author.

## References

[1] Wei H., Zhao G., Liu H., Wang H., Ni W., Wang P., Zheng Z. (2018). A simple and efficient method for the extraction and separation of menaquinone homologs from wet biomass of Flavobacterium. Bioproc. Biosyst. Eng..

[2] Sun J., Rao B., Zhang L., Shen Y., Wei D. (2012). Extraction of acetoin from fermentation broth using an acetone/phosphate aqueous two-phase system. Chem. Eng. Commun..

[3] Bhalerao S., Clandinin T. (2012). Vitamin K_2_ takes charge. Science.

[4] Vos M., Esposito G., Edirisinghe J., Vilain S., Haddad D., Slabbaert J., Van Meensel S., Schaap O., De Strooper B., Meganathan R. (2012). Vitamin K_2_ is a mitochondrial electron carrier that rescues Pink1 deficiency. Science.

[5] Szterk A., Zmysłowski A., Bus K. (2018). Identification of cis/trans isomers of menaquinone-7 in food as exemplified by dietary supplements. Food Chem..

[6] Xu X., Liu Z., Li H., Zhang X., Shi J., Chen J., Duan Z. (2021). Mutation and process optimization of Bacillus natto ND-1 for vitamin K2 (MK-7) production. Chin. J. Bioproc. Eng..

[7] Fang Z., Wang L., Zhao G., Liu H., Wei H., Wang H., Ni W., Zheng Z., Wang P. (2019). A simple and efficient preparative procedure for menaquinone-7 from Bacillus subtilis (natto) using two-stage extraction followed by microporous resins. Process Biochem..

[8] Chatake T., Yanagisawa Y., Inoue R., Sugiyama M., Matsuo T., Fujiwara S., Ohsugi T., Sumi H. (2018). Purification and structural characterization of water-soluble menaquinone-7 produced by Bacillus subtilis natto. J. Food Biochem..

[9] Zelazinski T., Sloma J., Skudlarski J., Ekielski A. (2020). The rape pomace and microcrystalline cellulose composites made by Press processing. Sustainability.

[10] Awal A., Rana M., Sain M. (2015). Thermorheological and mechanical properties of cellulose reinforced PLA bio-composites. Mech. Mater..

[11] Lisuzzo L., Caruso M., Cavallaro G., Milioto S., Lazzara G. (2021). Hydroxypropyl cellulose films filled with halloysite nanotubes/wax hybrid microspheres. Ind. Eng. Chem. Res..

[12] Zou J., Zhu H., Wang F., Sui H., Fan J. (2011). Preparation of a new inorganic–organic composite flocculant used in solid–liquid separation for waste drilling fluid. Chem. Eng. J..

[13] Liu T., Ding E., Xue F. (2017). Polyacrylamide and poly (*N*,*N*-dimethylacrylamide) grafted cellulose nanocrystals as efficient flocculants for kaolin suspension. Int. J. Biol. Macromol..

[14] Hong K., Wang Q., Chen J., Zang Y., Wang L., Liu N., Yue W., Nie G. (2020). Modification and application of cellulose. Sci. Technol. Food Ind..

[15] Wang H., He Y., He W., Wang Y., Wang R. (2012). Modification of cellulose and its application in wastewater treatment. Technol. Water Treat..

[16] Yan L., Tao H., Bangal P. (2010). Synthesis and flocculation behavior of cationic cellulose prepared in a NaOH/urea aqueous solution. Clean.

[17] Zhang H., Guo H., Wang B., Xiong L., Shi S., Chen X. (2016). Homogeneous synthesis and characterization of polyacrylamide-grafted cationic cellulose flocculants. J. Appl. Polym. Sci..

[18] Parviainen H., Hiltunen M., Maunu S. (2014). Preparation and flocculation behavior of cellulose-g-PMOTAC copolymer. J. Appl. Polym. Sci..

[19] Wei W. (2015). Preparation of Acrylic or Acrylamide Copolymer and Its Precipitation Research on Processing of Water-Soluble Organic Pollutants. Master’s Thesis.

[20] Wu Y., Zhang H., Zeng K., Lin Y., Zheng Z., Ding X. (2018). Oil-absorbing polymers from poly(lauryl methacrylate). Chem. Res. Appl..

[21] Duan Y., Bian F., Huang H. (2015). Facile fabrication of porous oil- absorbent microspheres with high oil absorbency and fast oil absorption speed. Polym. Adv. Technol..

[22] Dou Y., Li X., Liu G., Xu L. (2019). Determination of 2-methylnaphthalene and β-menthoquinone in reaction liquid by high performance liquid chromatography with external standard. Phys. Chem. Anal..

[23] Su Y., He H., Ming X., Zhou L. (2019). Determination of squalene in human skin cuticle by high performance liquid chromatography. G-Chem. Ind..

[24] Yan F., Shen Y., Ma G., Yang K. (2018). Synthesis and properties of polyacrylate high oil absorption resin. Fine Chem..

[25] Xie M., Chen Q., Yu J., Ding F., Chen L. (2011). Synthesis and characterization of styrene-lauryl methacrylate-acrylic acid copolymer. Fine Spec. Chem..

[26] Ren K., Sheng J., Chen J., Qi W., Guan X., Lin J. (2019). Modification of microcrystalline cellulose for preparing Cu2+ adsorption materials. J. For. Environ..

[27] Zhu H., Zhang Y., Yang X., Liu H., Shao L., Zhang X., Yao J. (2015). One-step green synthesis of non-hazardous dicarboxyl cellulose flocculant and its flocculation activity evaluation. J. Hazard. Mater..

[28] Zhang J., Sun L., Xiu Z. (2008). Removal of Klebsiella pneumoniae cells from 2,3-butanediol fermentation supernatant by flocculation and reuse of cells in flocs. Chin. J. Proc. Eng..

[29] Yang G., Zhang H., Zhang D., Cao Q., Yang J., Ji L., Mao Z. (2018). Cancer-specific chemotherapeutic strategy based on the vitamin K3 mediated ROS regenerative feedback and visualized drug release in vivo. Biomaterials.

[30] Zhu Y. (2019). Content of squalene in vegetable oils and its change during oil processing and utilization. Chin. Oils Fats.

